# Perioperative considerations for spinal cord stimulation devices: A practical guide^☆^

**DOI:** 10.1016/j.inpm.2025.100648

**Published:** 2025-10-29

**Authors:** Dorsa Kavandi, Eileen T. Jin, Salim M. Hayek, David Hao

**Affiliations:** a Department of Anesthesiology, Mass General Brigham, Harvard Medical School, Boston, MA, USA; b University Hospitals Cleveland Medical Center, Case Western Reserve University School of Medicine, Cleveland, OH, USA

**Keywords:** Neuromodulation, Implanted devices, Spinal cord stimulator

## 1 Introduction

As indications for neuromodulation continue to expand, spinal cord stimulators (SCS) are increasingly encountered in the perioperative setting. Previous efforts have provided valuable aggregation of management recommendations, compiled from diverse manufacturer sources [1,2]. This editorial builds upon this foundation by offering perioperative clinicians a concise and systematic approach that emphasizes actionable steps to preserve device integrity and ensure patient safety. Clinicians are advised to confirm recommendations through direct manufacturer consultation [2].

**Step 1: identify the device early**

**Fig. 1:**
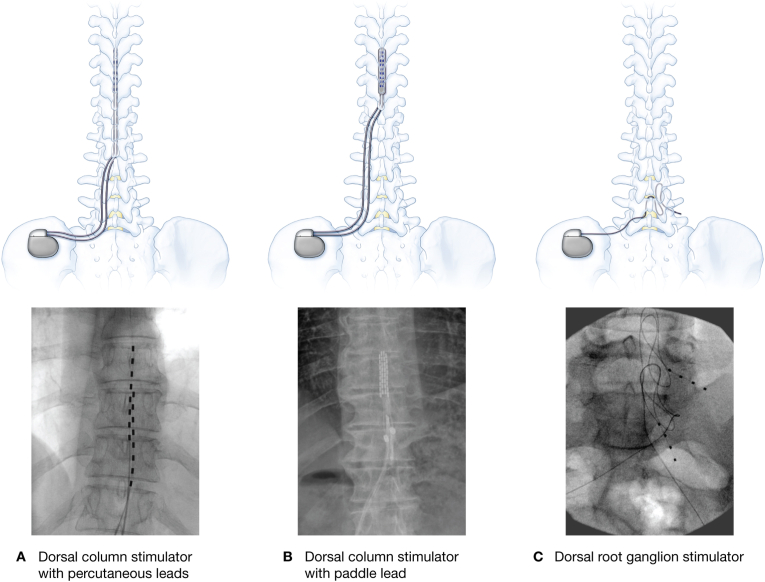
Representative types of spinal cord stimulators.

Early identification of SCS devices is critical for safe perioperative management [2]. Ideally, this evaluation should be completed before the patient arrives for the procedure (Fig. 1). During the preoperative assessment, clinicians should directly inquire about and document the device type and manufacturer, clinical indication, anatomical location of electrode(s) and implantable pulse generator (IPG), current operational status, and contact information for device representatives or the implanting pain management team. If there is uncertainty regarding the type of device (e.g., peripheral nerve stimulator vs. SCS) or the anatomical location of the leads, a plain film X-ray can be a helpful initial tool to clarify device characteristics.

If necessary, initiate contact with the implanting team, manufacturer, or local device representative well in advance of surgery [1]. Last-minute device recognition can lead to potential missed safety precautions, as manufacturer recommendations may vary. For example, some devices offer dedicated surgical modes such as Abbott's “surgery mode” [2].

**Step 2: power down the device**

For most SCS systems, the device should be turned off before the procedure [3]. Abbott systems require activation of “surgery mode” via the patient's controller or with assistance from a device representative [2]. Again, early device identification is crucial, as patients must bring their appropriate controllers to deactivate their devices. If the patient forgets their controller, a local representative may need to be contacted to turn off the device, potentially causing significant delays. Always confirm successful deactivation with the patient or device support team before proceeding.

In select situations where the patient does not have their controller, some systems may respond to an external magnet applied over the IPG for 10–15 seconds, which can deactivate the device in a manner similar to implanted cardioverter defibrillators. Because responses vary by manufacturer and device model, consultation with the device manufacturer or a specialist is recommended before considering this approach.

**Step 3: position carefully**

Care should be taken to minimize mechanical pressure on the implanted pulse generator (IPG). Identifying the IPG location prior to positioning allows for protective strategies, such as targeted padding. Although most IPGs are implanted in the posterolateral flank or buttock, implantation sites can vary. In patients with a recent SCS implant, particularly within the first six weeks, special attention should be given during positioning to avoid excessive spinal flexion, extension, or rotation, as these movements may increase the risk of lead migration [4].

**Step 4: electrocautery precautions**

Electrocautery poses a potential risk of malfunction or damage to implanted devices and leads [5]. All major neurostimulation device manufacturers recommend avoiding monopolar cautery whenever possible. Bipolar cautery is preferred, as it confines the electrical current between the two tips of the instrument and minimizes current propagation through the implanted system [3,6].

If monopolar electrocautery is unavoidable, precautions should be taken to reduce the risk of adverse events. Use the lowest effective current, favor low-voltage settings and apply short, intermittent bursts rather than continuous activation. The electrocautery return pad should be placed at least 10 cm from the IPG and, ideally, contralateral to both the IPG and electrodes to reduce the chance of current paths crossing the IPG or leads [1]. Another option is the use of a pulsed radiofrequency electrosurgical appliance. Such devices have been shown in both simulated and clinical settings to be safe around neuromodulation devices, facilitating surgical dissection [7,8].

**Step 5: neuraxial anesthesia, caution, not contraindication**

Epidural and spinal anesthesia are not absolute contraindications in patients with implanted stimulators; however, careful planning and individualized risk assessment are essential. The main concerns include the risk of direct lead trauma and block failure due to altered epidural space anatomy.

If considering neuraxial anesthesia, it is important to review radiologic images such as X-rays or CT scans to assess device configuration and lead trajectory. Given the potential for serious complications, neuraxial anesthesia should be avoided whenever feasible. Lead placement, anchoring, and the tunneling path vary by patient, so safety cannot be assumed based solely on the stimulator type (e.g., dorsal column or dorsal root ganglion). Detailed knowledge of the device configuration is critical before proceeding.

If a neuraxial block is deemed necessary, consultation with a pain specialist should be strongly considered. When performing the procedure, the needle must be inserted at a site that avoids the leads, anchors, and tunneled segments. Because this is difficult to ensure without fluoroscopy, live imaging is strongly recommended. Ultrasound may help identify the intended neuraxial level, but its utility is often limited in visualizing tunneled segments or anchors. Catheter placement adds further risk, as epidural catheters inserted with a landmark approach can unpredictably track in the epidural space. This risk is particularly relevant for dorsal root ganglion systems, where epidural tension relief loops may extend several levels above or below the actual lead location [1,2,4]. Another reason to be cautious is the risk of introducing an infection in the epidural space, especially in the setting of an indwelling catheter.

**Step 6: postoperative device reactivation**

Postoperative management should include confirmation of device function. For most systems, this involves turning the device back on and verifying that the settings match the preoperative configuration. For Abbott devices, specifically, “surgery mode” should be exited to restore prior functionality [2]. Routine interrogation in the recovery area is not required; however, if there is any loss of therapy or analgesia, the device should be evaluated promptly by the pain management team or a device manufacturer representative to identify and address potential malfunctions [4]. There is limited current evidence to guide the management of patients with concurrent implanted devices such as spinal cord stimulators, deep brain stimulators, and cardiac implantable electronic devices. It is reasonable to apply device-specific protocols and perform individualized risk assessment and monitoring. Prior literature has specifically reviewed the interaction between spinal cord stimulators and cardiac pacemakers (CPMs) or implantable cardioverter-defibrillators (ICDs), with no significant interference observed [9].

## Declaration of competing interest

The authors declare that they have no known competing financial interests or personal relationships that could have appeared to influence the work reported in this paper.
